# Sickle Retinopathy in a Person with Hemoglobin S/New York Disease

**DOI:** 10.1155/2012/136582

**Published:** 2012-12-31

**Authors:** Donovan Calder, Maryse Etienne-Julan, Marc Romana, Naomi Watkins, Jennifer M. Knight-Madden

**Affiliations:** ^1^Department of Surgery, Radiology, Anaesthetics, and Intensive Care, Faculty of Medical Sciences, University of the West Indies, Mona, Kingston 7, Jamaica; ^2^Universite des Antilles et de la Guyane, Centre Hospitalier Universitaire (CHU), Pointe-a-Pitre, UMR S 458 Inserm, 97159 Guadeloupe, France; ^3^Inserm U763, Pointe-à-Pitre, 97159 Guadeloupe, France; ^4^Université des Antilles et de la Guyane, 97159 Guadeloupe, France; ^5^Sickle Cell Unit, Tropical Medicine Research Institute, University of the West Indies, Mona, Kingston 7, Jamaica

## Abstract

A patient who presented with sickle retinopathy and hemoglobin electrophoresis results compatible with sickle cell trait was found, on further investigation, to be a compound heterozygote with hemoglobin S and hemoglobin New York disease. This recently reported form of sickle cell disease was not previously known to cause retinopathy and surprisingly was observed in a non-Asian individual. The ophthalmological findings, the laboratory diagnosis, and possible pathophysiology of this disorder are discussed. Persons diagnosed with sickle cell trait who present with symptoms of sickle cell disease may benefit from specific screening for this variant.

## 1. Introduction

Hemoglobin (Hb) New York, also known as HbK Kaohsiung, has been described in persons of Asian descent in New York, Asia [1–4], and Costa Rica [5] but has not previously been reported in the English speaking Caribbean. In combination with other abnormal globins, Hb New York has been implicated in clinical disorders such as severe HbH syndrome [6] and cholelithiasis [4]. Hemoglobin S/New York disease was recently reported as a clinically significant type of sickle cell disease (SCD), causing splenic disease in two patients as well as Acute Chest Syndrome (ACS) in one of them [7]. Sickle cell disease develops in individuals who carry two significant mutations in the beta (*β*) globin allele, at least one of which must result in the production of hemoglobin S. The phenotype of persons with SCD varies depending on the second allele. Severe genotypes include homozygous SS disease and hemoglobin S*β*° thalassemia disease whereas mild genotypes include hemoglobin SC disease and hemoglobin S*β* ^+^ thalassemia disease. Our patient who presented with sickle retinopathy and a vitreous haemorrhage was found to have hemoglobin S/New York disease. The ophthalmological findings, the laboratory diagnosis, and possible pathophysiology of this disorder are discussed.

Ethical approval from the Faculty of Medical Sciences/University Hospital of the West Indies Ethical Committee and written informed consent were obtained. A complete ophthalmologic examination was performed. We defined sickle cell retinopathy as any salmon patch hemorrhages, iridescent spots, black sunbursts, retinal neovascularization, or retinal detachment.

## 2. Case Presentation

**Figure 1:**
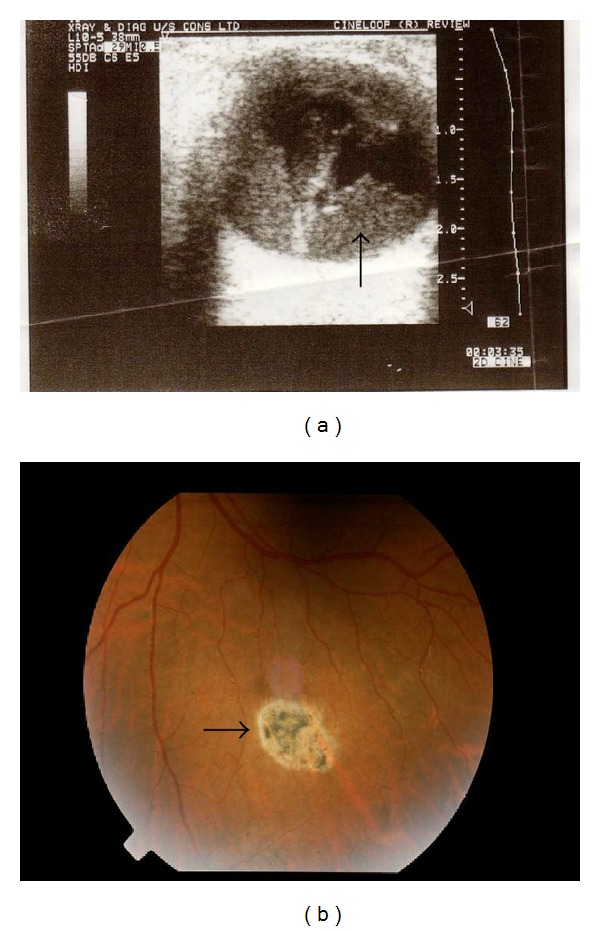
(a) Ultrasound image of the left eye of the patient demonstrating vitreous hemorrhage. (b) Black starburst in peripheral retina of left eye.

The patient, a 48-year-old male, presented with a one week history of poor vision from the left eye. The ocular history was one of myopia and the medical history was notable for a history of mild hypertension. Visual acuities were best corrected to 20/20 on the right and hand movement on the left. Fundoscopy of the right eye was unremarkable for hypertensive retinopathy or sickle retinopathy whereas the left fundal view was obscured on the left by a vitreous hemorrhage (Figure 1(a)). The vitreous hemorrhage cleared over a year leaving behind only a pigmented “Black Starburst” on fundoscopy (Figure 1(b)). These findings, while not in keeping with hypertensive retinopathy are typical of sickle retinopathy.

Initial diagnostic tests performed at the Sickle Cell Unit included hemoglobin status by citrate agar and cellulose acetate electrophoresis and complete blood count by an automated cell counter. DNA samples and blood samples were sent to Guadeloupe where isoelectrofocusing and cation-exchange high performance liquid chromatography were performed on the blood sample. Genomic DNA, obtained from peripheral blood, was extracted by standard phenol-chloroform procedure and the sickle genotype was established by DNA sequencing of the *β*-globin gene coding region. The amplified fragments were column purified and cycle sequenced with the ABI PRISM Big Dye Termination ready reaction kit and analyzed by an ABI 310 DNA sequencer (PE Applied Biosystems, Foster city, USA). 

**Figure 2:**
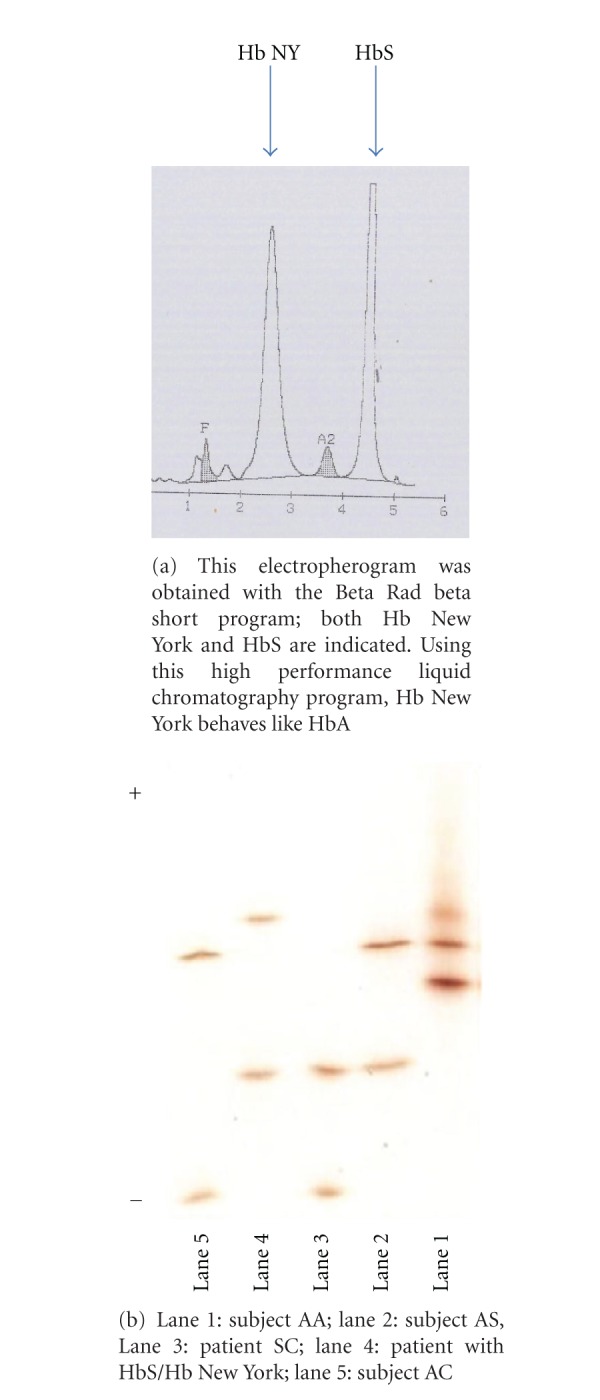
Electrophoresis data of the patient HbS/Hb New york.(a) Electropherogram of the blood sample of the patient with HbS/Hb New York Disease. (b) Isoelectrofocusing data for the same sample.

The hemoglobin (13.7 g/dL), citrate and cellulose acetate agar electrophoresis were consistent with sickle cell trait (SCT) (Figure 2(a)). Isoelectric focusing identified sickle protein and an unknown protein (Figure 2(b)). Sequencing of promoter region, exons 1, 2, and 3 revealed mutations as follows: HBB c.20A>T (HbS), HBB c.341T>A (Hb New York).

## 3. Discussion

Hemoglobin New York, also known as HbK Kaohsiung, has been described most often in persons of Asian descent. Similar to the two patients previously described, [7] this patient is not aware of having Asian ancestry. Unlike the previously reported patients, the patient we report had retinopathy. This further suggests that hemoglobin S/New York disease may be a clinically significant form of SCD, causing diverse complications. The interaction between Hb New York and HbS may be significant due to the properties of Hb New York, which though difficult to distinguish from HbA by electrophoresis [6] or HPLC [8], like HbS has decreased oxygen affinity compared with HbA [9]. Patients with SCT rarely develop sickle retinopathy [10]. Persons diagnosed as SCT who present with symptoms of SCD may benefit from specific screening for this variant.
